# Major Aortopulmonary Collateral Artery as a Rare Cause of Heart Failure in a Neonate with Transposition of the Great Arteries: An Unexpected Turn in an Otherwise Routine Case

**DOI:** 10.3390/jcdd13050210

**Published:** 2026-05-14

**Authors:** Vojislav Parezanovic, Dusan Andric, Vladimir Chadikovski, Vedran Stojanovikj, Jordanka Madzoska, Vesna Trpkovska, Igor Stefanovic

**Affiliations:** 1 Department of Cardiology, University Children’s Hospital, 11000 Belgrade, Serbia; 2 Medical Faculty, University of Belgrade, 11000 Belgrade, Serbia; 3 Department of Pediatric Cardiac Surgery, Acibadem Sistina Hospital, 1000 Skopje, North Macedoniajordanka.madzoska@acibademsistina.mk (J.M.);

**Keywords:** transposition of the great arteries, major aortopulmonary collateral, arterial switch operation, vascular coil

## Abstract

The association of a major aortopulmonary collateral artery (MAPCA) with simple transposition of the great arteries (TGA) is uncommon. Such high-flow lesions in the postoperative period following arterial switch operation (ASO) may lead to pulmonary hypertension, pulmonary hemorrhage, heart failure (HF), failure to thrive and prolonged mechanical ventilation. We report a neonate who developed pulmonary overcirculation and HF in the early postoperative period due to a hemodynamically significant MAPCA. Although the association of MAPCA with simple TGA is infrequent, such lesions should be considered in cases of unexplained cardiovascular compromise following ASO. Following transcatheter occlusion of the MAPCA with a vascular coil, rapid hemodynamic stabilization and subsequent extubation of the patient were achieved.

## 1. Introduction

Simple transposition of the great arteries (TGA) is a congenital heart defect defined by ventriculoarterial discordance in the setting of atrioventricular concordance, without major associated intracardiac or extracardiac anomalies. In this condition, the morphologic right ventricle gives rise to the aorta, while the morphologic left ventricle gives rise to the pulmonary artery, resulting in parallel rather than serial circulation. This physiology is incompatible with life unless adequate mixing between the systemic and pulmonary circulations is present, most commonly at the level of a patent ductus arteriosus or an atrial septal defect [1]. MAPCAs are blood vessels originating from the aorta or its major branches that supply a portion of the pulmonary vasculature. Embryologically, they represent persistent segmental arteries. They are most commonly observed in association with duct-dependent pulmonary circulation and severe cyanotic congenital heart anomalies [2]. MAPCAs associated with TGA are uncommon and usually represent a silent lesion. However, when hemodynamically significant, they may become symptomatic and lead to an unexpectedly complicated postoperative course. Such a high-flow lesion may result in left-to-right shunting, pulmonary overcirculation, pulmonary hemorrhage or pulmonary hypertension, left ventricle volume overload with HF and prolonged mechanical ventilation [3].

## 2. Case Report

### 2.1. Preoperative Findings

The neonate was born at term, at 38 + 0 weeks of gestation, via vaginal delivery, with a birth weight of 3030 g and birth length of 50 cm. The Apgar scores were 8 and 9 at 1st and 5th minutes of life, respectively. The diagnosis of simple TGA was established prenatally. Postnatal echocardiography confirmed TGA without evidence of additional intracardiac or extracardiac structural abnormalities. Adequate mixing was present at the atrial level through a non-restrictive interatrial communication, with additional but limited contribution from a patent ductus arteriosus that was already showing signs of constriction. Immediately after birth, low peripheral oxygen saturation of about 60% was recorded. Due to pronounced tachydyspnea, the patient was intubated with a 3.5 mm endotracheal tube and placed on mechanical ventilation in PC-SIMV mode. A continuous infusion of PGE_1_ was initiated at a dose of 10 ng/kg/min, which was gradually reduced to 5 ng/kg/min. During the preoperative period, the patient remained hemodynamically stable but developed two episodes of junctional ectopic tachycardia with a heart rate of 250/min, which were successfully converted with amiodarone. Invasively measured arterial blood pressure was 65/30 mmHg. Oxygen saturation ranged from 70 to 82%. Therefore, Rashkind balloon atrial septostomy was not performed.

### 2.2. Surgical Management

On the 7th day of life, a cardiac surgical intervention was performed, consisting of an ASO with Lecompte maneuver and ligation of the patent ductus arteriosus. The aortic cross-clamp time was 99 min, and the total cardiopulmonary bypass time was 161 min. The patient left the operating room on vasopressor with epinephrine at a dose of 0.01 µg/kg/min.

### 2.3. Postoperative Complications and Management

Due to hemodynamic instability, the chest was closed on the 1st postoperative day. The neonate began to tolerate minimal enteral feeding but became oliguric. Clinical examination revealed generalized subcutaneous edema, predominantly facial and pretibial, along with hepatomegaly. Chest X-ray demonstrated prominent pulmonary vascularity. Echocardiographic re-evaluation revealed impaired left ventricular systolic function, moderate atrioventricular valve regurgitation, signs of pulmonary hypertension and a dilated inferior vena cava with reduced collapsibility. Continuous diuretic stimulation with furosemide (0.2 mg/kg/h) was initiated, along with renal-dose dopamine (3 µg/kg/min). Epinephrine was discontinued and due to signs of low cardiac output syndrome, inodilator support with milrinone was initiated at a dose of 0.5 µg/kg/min. After three days, the edema regressed and urine output improved, allowing discontinuation of inotropic support with dopamine and milrinone. Continuous diuretic therapy with furosemide was maintained, and spironolactone (3 mg once daily) was added enterally. Despite significant clinical improvement in the following days, the patient experienced several unsuccessful extubation attempts accompanied by episodes of hypercapnic respiratory failure. To maintain an adequate ventilation–perfusion ratio, relatively high levels of PEEP (positive end-expiratory pressure) were required (up to 7.0 cmH_2_O). On repeat echocardiographic reevaluation, signs of pulmonary hypertension persisted. From the suprasternal views, a continuous flow profile suggestive of a high-flow lesion was detected in continuity with the aortic arch. A MAPCA was suspected, and diagnostic cardiac catheterization was therefore performed.

### 2.4. Diagnostic and Interventional Treatment

**Figure 1:**
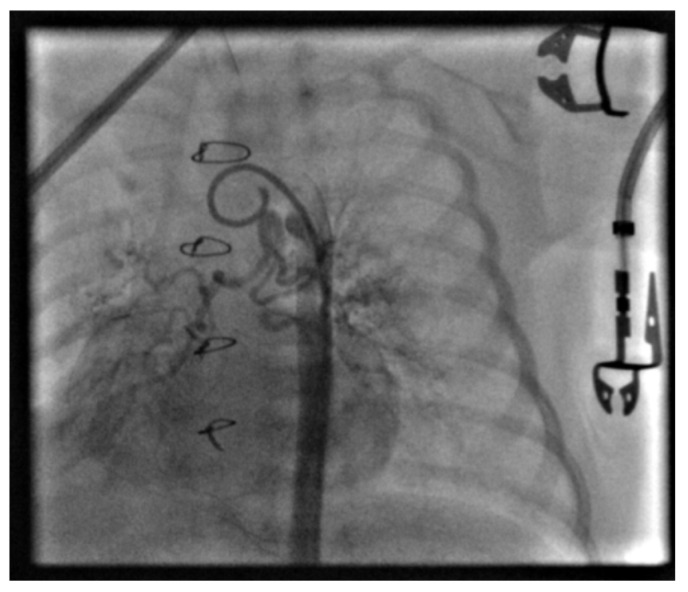
Aortography indicates a hemodynamically significant MAPCA and a rapid contrast washout phenomenon.

After standard preparation, under general endotracheal anesthesia, left heart catheterization was performed on the 34th day of life. The right femoral artery was punctured and a pigtail catheter was advanced through the abdominal and thoracic aorta to the aortic arch, where aortography was performed. (Figure 1).

**Figure 2:**
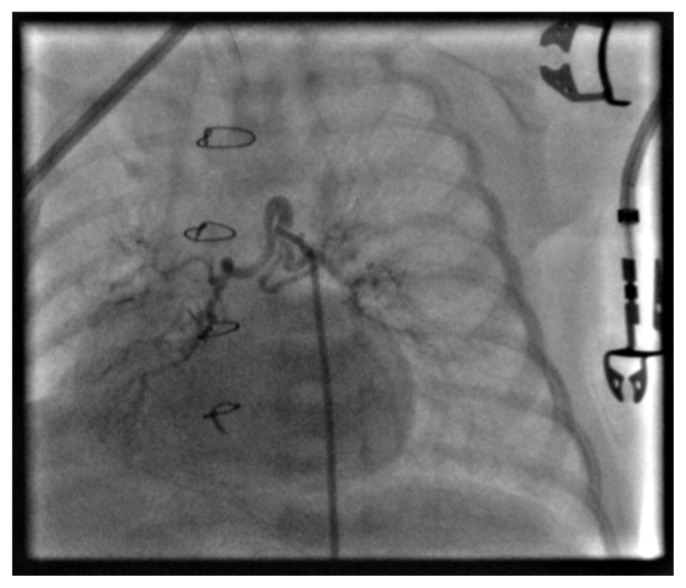
Selective angiography of the MAPCA demonstrates its branching into three smaller collaterals, of which the largest supplies the right pulmonary lobe, while the other two supply the left pulmonary lobe.

During the aortography, rapid contrast washout with early opacification of the lung fields was observed, raising strong suspicion of a hemodynamically significant MAPCA (hsMAPCA). A MAPCA originating from the descending aorta was identified. The proximal vessel diameter measured approximately 2 mm. The collateral coursed superiorly, forming a downward loop, and subsequently divided into three branches. The largest branch, measuring 1.6 mm in diameter, supplied the right lung, while the remaining two branches, measuring approximately 1–1.3 mm, supplied the left lung. (Figure 2).

**Figure 3:**
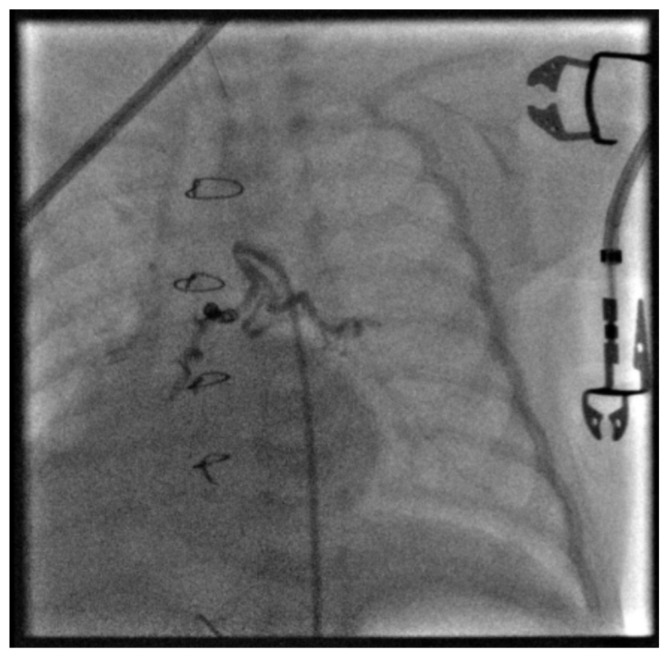
An Azur CX Vascular Coil 3 × 4 mm was placed in the distal, largest branch of the MAPCA supplying the right pulmonary lobe. Due to its very distal position, contrast opacification of the left pulmonary lobe is still observed.

A decision was made to proceed with transcatheter occlusion of the collateral using Azur CX coils (Terumo, Tokyo, Japan). A Progreat microcatheter (Terumo, Tokyo, Japan) was advanced over a guidewire into the distal segments of the MAPCA. After removal of the guidewire, a 3 mm × 4 mm coil was carefully deployed in the larger branch supplying the right lung. (Figure 3).

**Figure 4:**
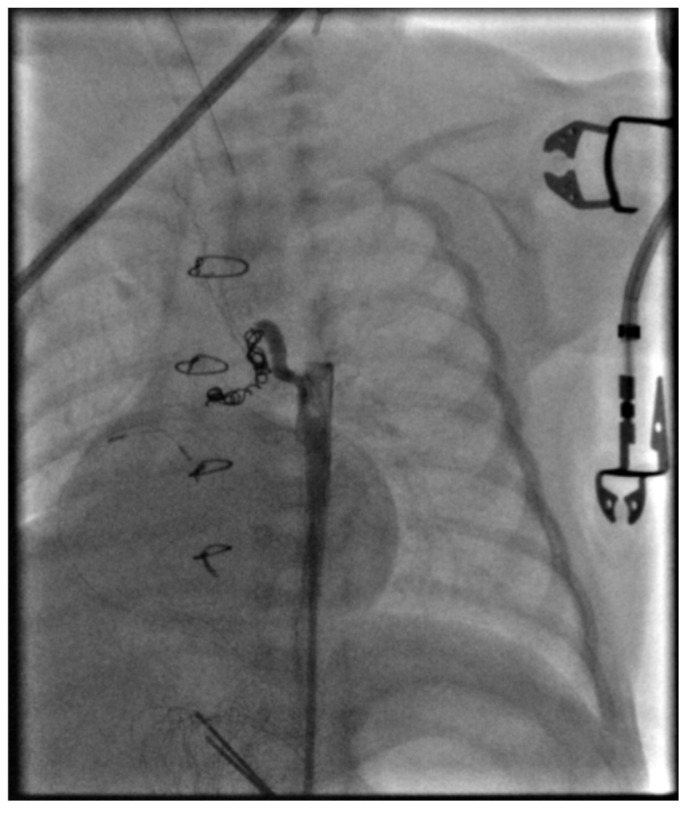
Two additional coils were placed proximal to the landing zone of the first coil. All three branches of the MAPCA are now occluded, and control angiography shows no residual flow.

Subsequently, two additional coils were deployed proximally relative to the first coil, covering the origins of the other two collateral branches supplying the left lung (Figure 4).

Control selective angiography through the main collateral trunk demonstrated appropriate coil positioning with complete occlusion of the collateral vessel. Direct manometry revealed an aortic pressure of 68/28/41 mmHg (systolic/diastolic/mean) before occlusion and 74/42/52 mmHg after occlusion. This finding strongly suggests the presence of diastolic runoff prior to the intervention, as the diastolic pressure increased by 14 mmHg following MAPCA occlusion. Another phenomenon associated with diastolic runoff is a widened pulse pressure, measuring 40 mmHg before occlusion and 32 mmHg afterward.

No procedural complications occurred apart from transient rhythm disturbances, including supraventricular extrasystoles and brief episodes of supraventricular tachycardia.

### 2.5. Outcome and Follow-Up

The neonate was successfully extubated the day after the intervention and remained on oral furosemide and spironolactone. The patient was discharged home two weeks later in good clinical condition and without symptoms, and follow-up was initiated.

## 3. Discussion

### 3.1. MAPCA Classification, Pathophysiology and Hemodynamics

MAPCAs represent a rare congenital vascular anomaly, considered remnants of the fetal splanchnic circulation and are most commonly associated with cyanotic congenital heart anomalies with pulmonary hypoperfusion, such as Tetralogy of Fallot or pulmonary atresia [4]. In a retrospective cohort of 671 patients with simple TGA undergoing ASO, MAPCA were identified in 13 cases (1.9%) [5]. Although the incidence of hsMAPCA is very low, they should be considered in cases of a complicated postoperative course after ASO, particularly when heart or respiratory failure occurs.

According to their origin, MAPCA can be divided into three types: type I arising indirectly from bronchial arteries, type II directly from the aorta, and type III indirectly from aortic branches. When a MAPCA represents the sole feeding artery to a specific lung segment or lobe, it is considered essential (non-communicating). In contrast, when it supplies a segment or lobe alongside the corresponding pulmonary arterial branch, it is classified as redundant (communicating). MAPCAs may also be classified as hypertensive or non-hypertensive based on the presence of stenosis [6]. In our case, the MAPCA was classified as type II, non-hypertensive, originating from the descending thoracic aorta just below the carina and was redundant. The mechanism by which a MAPCA, as a high-flow lesion, leads to cardiovascular deterioration is through a left-to-right shunt. Following ASO, the physiological decrease in pulmonary vascular resistance after birth further augments the shunt volumes; higher levels of PEEP were required to keep the alveoli open and allow adequate gas exchange. This pathophysiological “steal” toward the pulmonary circulation leads to pulmonary overcirculation and compromised systemic blood flow. The main hemodynamic consequence is pulmonary hyperperfusion, accompanied by coronary and systemic hypoperfusion [7]. Consequently, the patient developed myocardial stunning with concomitant left heart volume overload, resulting in low cardiac output syndrome and oliguria. During cardiac surgery, a MAPCA may be suspected in the presence of increased left atrial venous return. In our case, as no atriotomy was performed, direct assessment was not possible, and excessive pulmonary venous inflow was not recognized intraoperatively. Despite initially successful surgery, the combination of repeated failed extubation attempts, persistently high mechanical ventilation requirements, low cardiac output syndrome, and pulmonary plethora on chest X-ray raised a strong suspicion of an underlying MAPCA.

### 3.2. Transcatheter Occlusion Technique and Device Selection

**Figure 5:**
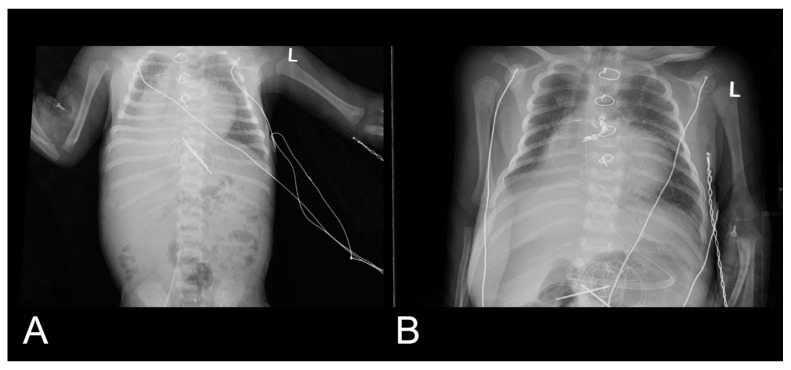
(**A**) Chest X-ray demonstrating increased pulmonary vascular markings, particularly in the right lung fields, consistent with pulmonary plethora. The cardiothoracic index was 0.65. (**B**) Chest X-ray obtained one day after transcatheter occlusion of the MAPCA showing resolution of pulmonary plethora. The cardiothoracic index was closer to normal and measured 0.55. Median sternotomy wires and temporary epicardial pacing wires are visible.

Cardiac catheterization remains the gold standard for the accurate diagnosis of MAPCAs. It enables precise assessment of the MAPCA origin, its arborization, diameter, and the pulmonary territory it supplies. During diagnostic catheterization, if a hsMAPCA is confirmed, conversion to an interventional procedure can be performed rapidly and straightforwardly [8]. The initial aortography revealed early and rapid aortic washout, accompanied by prompt opacification of the respective pulmonary fields, suggesting significant systemic-to-pulmonary shunting. A continuous “steal phenomenon” occurs during diastole through the low-resistance MAPCA, resulting in diastolic runoff and consequently low diastolic blood pressure. Chest X-ray clearly demonstrates active pulmonary hyperemia prior to MAPCA closure. Following transcatheter occlusion of the MAPCA, a significant improvement in chest X-ray was observed (Figure 5).

We used the AZUR CX Hydrogel Peripheral Coil (Terumo), a hydrogel-based embolization device that expands after deployment, promoting mechanical vessel occlusion and stable anchoring while reducing the risk of migration. Compared with non-hydrogel coils, it provides more controlled occlusion and improved conformability to vessel anatomy. Hydrogel expansion upon contact with blood increases volumetric filling and packing density, facilitating rapid occlusion and reducing the likelihood of residual flow or recanalization. Coil size was selected using a standard oversizing technique (20–30% larger than the measured vessel diameter). Delivery was performed through a 0.021″ Progreat microcatheter, with controlled detachment using the AZUR Detachment Controller, allowing precise and safe deployment [5,9,10,11].

## 4. Conclusions

Although the association of hsMAPCA with simple TGA is a rare combination of congenital anomalies, it should be considered in cases of an unexplained complicated postoperative course. Such patients often require prolonged stays in the neonatal intensive care unit, frequently need inotropic support, and may require prolonged mechanical ventilation. Therefore, early diagnosis of MAPCA and timely management are essential. Postoperative transcatheter closure of MAPCA using vascular coils represents an effective treatment option for resolving HF in carefully selected patients.

## Data Availability

The original contributions presented in this study are included in the article material. Further inquiries can be directed to the corresponding author.

## Abbreviations

The following abbreviations are used in this manuscript:

ASO	arterial switch operation
HF	heart failure
hsMAPCA	hemodynamically significant major aortopulmonary collateral artery
MAPCA	major aortopulmonary collateral artery
PEEP	positive end-expiratory pressure
TGA	transposition of the great arteries
